# AUDA suppresses apoptosis of HCAECs induced by TNF and serum of Kawasaki disease patients by inhibiting endoplasmic reticulum stress

**DOI:** 10.1515/biol-2025-1254

**Published:** 2026-04-17

**Authors:** Na Dai, Qiaoling Wu, Jin Wang, Li Li

**Affiliations:** Department of Pediatrics, Jinan Maternity and Child Care Hospital Affiliated to Shandong First University, Jinan, Shandong, China

**Keywords:** Kawasaki disease, endoplasmic reticulum stress, AUDA, apoptosis, proliferation

## Abstract

Kawasaki disease (KD) is an acute autoimmune vasculitis and the most common cause of acute vasculitis and acquired heart disease in children. 12-(3-adamantan-1-yl-ureido)-dodecanoic acid (AUDA) was identified as a potential therapeutic drug for KD, although its mechanism remained unclear. HCAECs were treated with TNF and serum from patients with KD to construct a KD cell model. CCK8, Elisa, and flow cytometry were performed to evaluate cell function, and western blot was used to detect target proteins. TNF and KD serum induced endoplasmic reticulum stress (ERS) and apoptosis in HCAECs. AUDA alleviated ERS and apoptosis induced by TNF or KD serum, as well as the inhibition of cell viability. Mechanistically, STAT1 transcription enhancer (2-NP) 2-NP inhibited the promoting effect of AUDA on cell proliferation and blocked the inhibitory effect of AUDA on ERS in TNF or KD serum-treated HCAECs. AUDA inhibits TNF and KD serum-induced ERS and apoptosis. This study improves our understanding of the pathogenesis of KD and provides a potential theoretical basis for its treatment.

## 1 Introduction

Kawasaki disease (KD) is an acute autoimmune vasculitis that commonly occurs in children under the age of 5 years. The incidence rate of KD children in China ranks third worldwide. In developing countries, KD is the most common cause of acute vasculitis and acquired heart disease in children [1]. In China and the United States, KD is the primary cause of childhood-acquired heart disease [2], 3].

In our previous research, 12-(3-adamantan-1-yl-ureido)-dodecanoic acid (AUDA) was identified as a potential therapeutic drug for KD [4], [5], [6], [7]. AUDA has been proved to promote human coronary artery endothelial cells (HCAECs) activity, facilitate vascular repair, and alleviate inflammation in the heart tissue of KD mice through the Peroxisome proliferator-activated receptor gamma (PPARγ)/Signal transducer and activator of transcription 1 (STAT1) signaling pathway [4].

Vascular endothelial cells contain abundant endoplasmic reticulum (ER), which is involved in protein synthesis, calcium homeostasis, and regulation of apoptosis [8], 9]. ER stress (ERS) is a stress state caused by ER dysfunction, which is a direct cause of cellular aging, apoptosis, and KD progression [10], 11]. This study aims to elucidate the mechanism by which AUDA inhibits endoplasmic reticulum stress and apoptosis in KD via the STAT1 signaling pathway. We focused on the therapeutic effect of AUDA on KD-induced HCAECs ERS and apoptosis, exploring the inhibition of AUDA on KD vascular injury and the protective effect on vascular lesions. This study improves our understanding of the pathogenesis of KD and provides a potential theoretical basis for its treatment.

## 2 Methods

### 2.1 Cell culture and treatment

HCAECs were purchased from the Wuhan Culture Collection, and cultured as described in our previous study [12]. HCAECs were cultured in endothelial culture medium (ECM) with 5 % fetal bovine serum (FBS), 1 % penicillin/streptomycin and 1 % endothelial cell growth supplement (ScienCell Research Laboratories, Inc., San Diego, CA, USA) at 37 °C in 5 % CO_2_. TNF is a cytokine that induces apoptosis [13]. HCAECs were incubated with 1 μg/mL tumor necrosis factor (TNF) to generate KD cell model with PBS as a control. Serum from patients with KD and healthy donors was collected from the Jinan Maternity and Child Care Hospital. HCAECs were cultured in medium with 15 % serum from healthy donors or KD patients to generate the control or KD cell model, respectively. 45 mmol/L of STAT1 transcription enhancer (2-NP) (MedChemExpress, Monmouth Junction, NJ, USA) was used to activate the Janus kinase (JAK)/STAT1 signaling pathway.

### 2.2 Cell counting kit-8 (CCK8)

CCK8 assay (Solarbio Science & Technology, Beijing, China) was performed to detect the viability of HCAECs. HCAECs were incubated in a 96-well plate, and the viability was detected every 24 h. 10 μl of CCK8 reagent was added to the cells and incubated for 1.5 h. Optical density (OD) was detected using a microplate reader.

### 2.3 Elisa

ELISA kit for Lactate dehydrogenase (LDH) (Solarbio Science & Technology, Beijing, China) was used for the detection of LDH in each group. After treatment for 24 h, the cell supernatant was collected, and the LDH level was detected according to the manufacturer’s instructions [14]. The absorbance was measured at 490 nm using a microplate reader (FLUOstar Omega, BMG LABTECH, Offenburg, Germany).

### 2.4 Flow cytometry

Apoptosis of HCAECs seeded at 5 × 10^5^ cells/well in 6-well plates after 24 h of treatment was detected using the Annexin V-FITC/PI Kit (4A BIOTECH, Beijing, China) according to the manufacturer’s instructions. Collected cells were resuspended in binding buffer, and incubated with Annexin V/FITC and PI [15]. Cells were then analyzed by flow cytometry (BD FACSCanto II system, BD, Franklin Lakes, NJ, USA), and the apoptotic cells were analyzed using a FlowJo software.

### 2.5 Western blot

After treatment for 24 h, Cells were lysed using RIPA buffer (Solarbio Science & Technology, Beijing, China). The sample was separated via sodium dodecyl sulfate-polyacrylamide gel electrophoresis (SDS-PAGE) and transferred to a polyvinylidene fluoride (PVDF) membrane. After blocked with 5 % milk for 1 h, proteins were incubated with following antibodies: IRE1α (28164-1-AP, Proteintech Group, Chicago, IL, USA), XBP1 (ab37152, Abcam, Cambridge, UK), CHOP (15204-1-AP, Proteintech Group, Chicago, IL, USA), ATF6 (ab227830, Abcam, Cambridge, UK) and Cleaved-Caspase12 (ab315271, Abcam, Cambridge, UK), Caspase12 (ab315271, Abcam, Cambridge, UK), Bax (ab32503, Abcam, Cambridge, UK), Cleaved-Caspase3 (ab214430, Abcam, Cambridge, UK), Bcl2 (12789-1-AP, Proteintech Group, Chicago, IL, USA), Caspase3 (ab184787, Abcam, Cambridge, UK) and GAPDH (10494-1-AP, Proteintech Group, Chicago, IL, USA). Then, samples were incubated with secondary antibody for 1 h, followed by developed with ECL kit. The bands were observed by a gel imaging system and then analyzed using Image J software [16].

### 2.6 Statistical analysis

Data analysis was performed using Graphpad Prism 9. Comparison between groups was executed using analysis of variance (ANOVA) analysis. *P* value of <0.05 was considered statistically significant. All data were generated from 3 independent repeated experiments.

## 3 Results

### 3.1 AUDA alleviates TNF induced ERS in HCAECs

**Figure 1:**
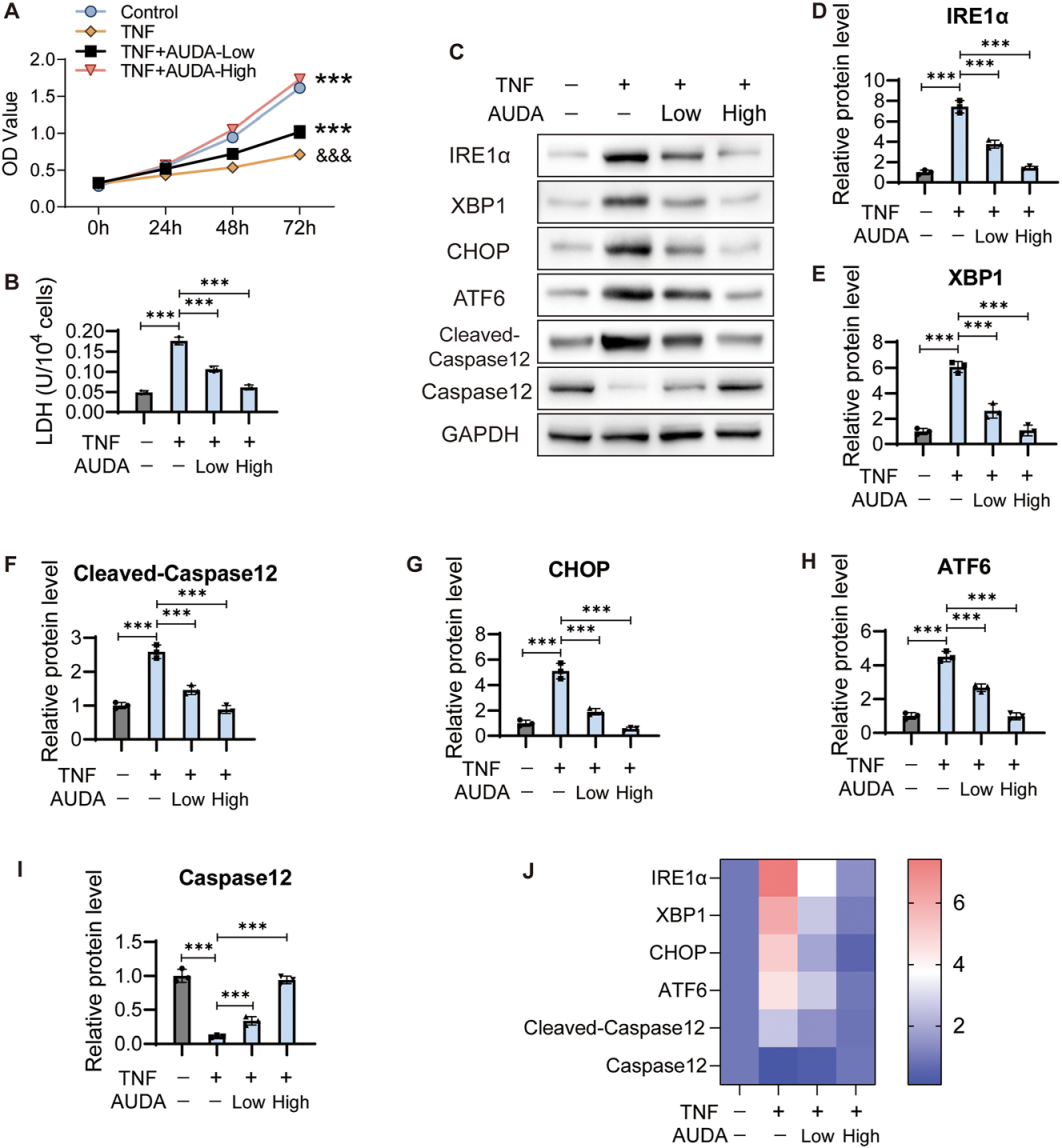
AUDA alleviates ERS induced by TNF in HCAECs. (A) HCAECs were treated with 1 μg/mL TNF to generate a KD cell model, with PBS as the control group. After treatment with TNF, HCAECs were treated with 50 μmol/L (low) and 100 μmol/L (high) AUDA for 24 h. Cell viability was detected using CCK8 assay. ***P < 0.001 versus control, &&&*P* < 0.001 versus TNF treated cells. (B) LDH level was detected using Elisa assay. (C) Expression of ERS marker factors was detected. The protein levels of IRE1α (D), XBP1 (E), CHOP (F), ATF6 (G), Cleaved-Caspase12 (H) and Caspase12 (I) were standardized to GAPDH. (J) Heatmap of ERS marker factors expression. ***P < 0.001.

To investigate the role of AUDA in KD cells, HCAECs were treated with 1 μg/mL TNF to generate a KD cell model with PBS as a control. Then, TNF treated HCAECs were incubated with 50 μmol/L (low) and 100 μmol/L (high) AUDA for 24 h. The viability of HCAECs treated with 1 μg/mL TNF was significantly decreased compared to of that the control cells, proving TNF-induced cell injury in HCAECs. After treatment with 50 μmol/L or 100 μmol/L AUDA, cell viability significantly increased (Figure 1A).

During ER stress, LDH is released. Cell supernatants were collected from each group, and LDH was detected using Elisa assay. This analysis revealed that TNF treatment increased the LDH level, and 50 μmol/L or 100 μmol/L AUDA markedly decreased the LDH level in HCAECs (Figure 1B). Based on the common characteristics of ERS, we expected that AUDA would inhibit ERS induced by TNF treatment.

As a potential consequence of severe ERS Cell death/cytotoxicity was evaluated by measuring LDH release. To specifically monitor ERS activation, we examined the expression of ERS-related proteins using western blot (Figure 1C). Indeed, the expression levels of IRE1α (Figure 1D), XBP1 (Figure 1E), CHOP (Figure 1F), ATF6 (Figure 1G) and Cleaved-Caspase12 (Figure 1H) were inhibited by 50 or 100 μmol/L AUDA in TNF treated HCAECs. In contrast, Caspase12 expression was enhanced by 50 μmol/L or 100 μmol/L AUDA in TNF treated HCAECs (Figure 1I).

### 3.2 AUDA inhibits TNF induced apoptosis in HCAECs

**Figure 2:**
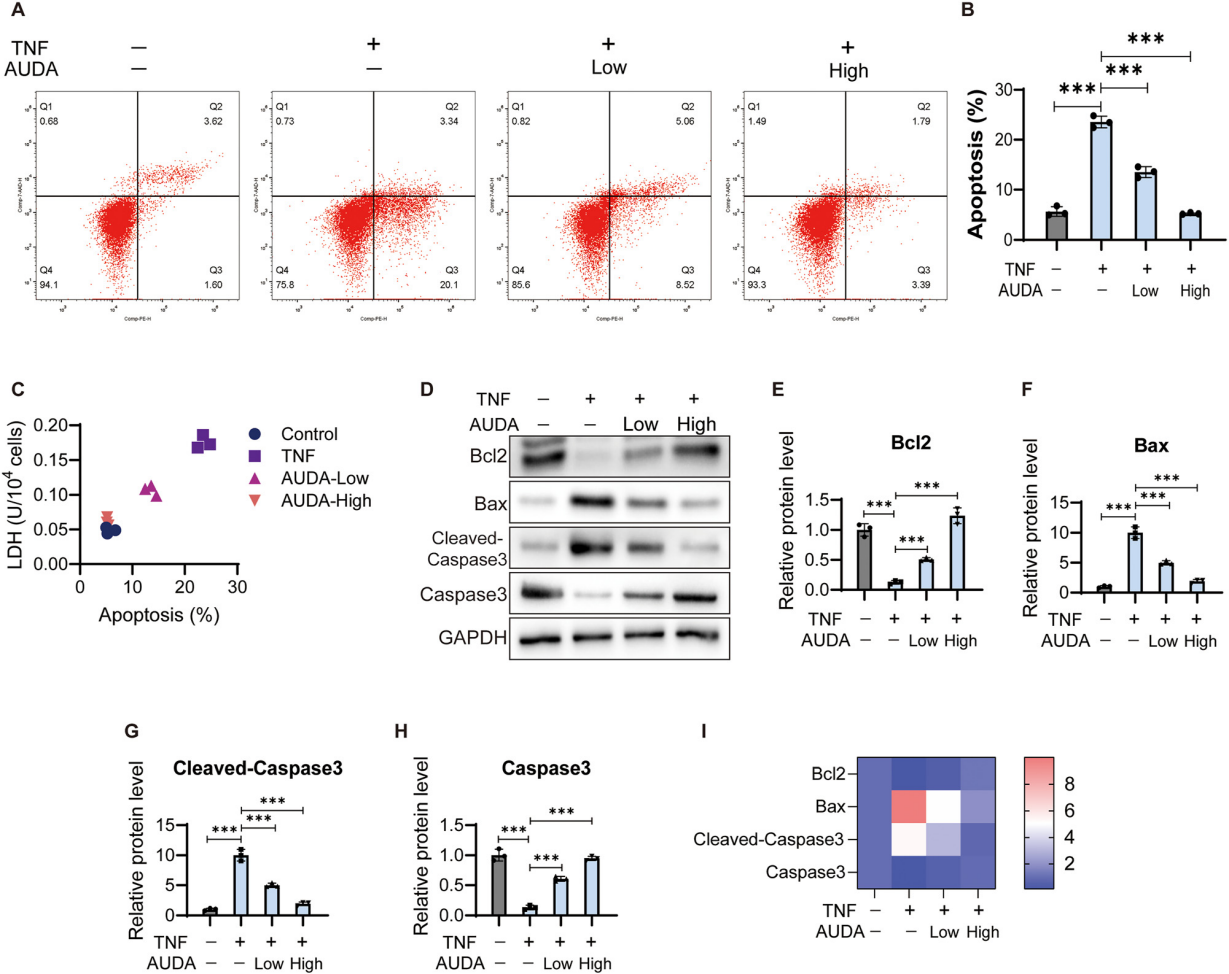
AUDA inhibits TNF induced apoptosis in HCAECs. (A) Apoptosis was analyzed using flow cytometry. (B) Percentage of apoptotic cells was analyzed on FlowJo software. (C) The correlation between apoptosis and LDH. (D) Apoptotic protein expression was detected. The protein levels of Bcl2 (E), Bax (F), Cleaved-Caspase3 (G) and Caspase3 (H) were standardized to GAPDH. (I) Heatmap of apoptotic protein expression. ***P < 0.001.

To further evaluate the effect of AUDA on KD cells, we detected the apoptosis of HCAECs in each group using flow cytometry (Figure 2A). The percentage of apoptotic cells increased significantly in TNF group, and decreased after AUDA treatment (Figure 2B). In addition, the trend in apoptosis was consistent with that of LDH across all groups (Figure 2C). This result proved that AUDA could inhibit TNF-induced apoptosis in HCAECs. To analyze the molecular mechanism, apoptosis-related proteins were detected using western blot (Figure 2D). TNF treatment increased the levels of Bax and Cleaved-Caspase3, while decreasing the levels of Bcl2 and Caspase3 (Figure 2E–I). After treatment with AUDA for 24 h, Bax and Cleaved-Caspase3 expression was inhibited, and Bcl2 and Caspase3 expression was significantly promoted compared with the TNF group cells (Figure 2E–I).

### 3.3 AUDA alleviates ERS induced by KD serum in HCAECs

**Figure 3:**
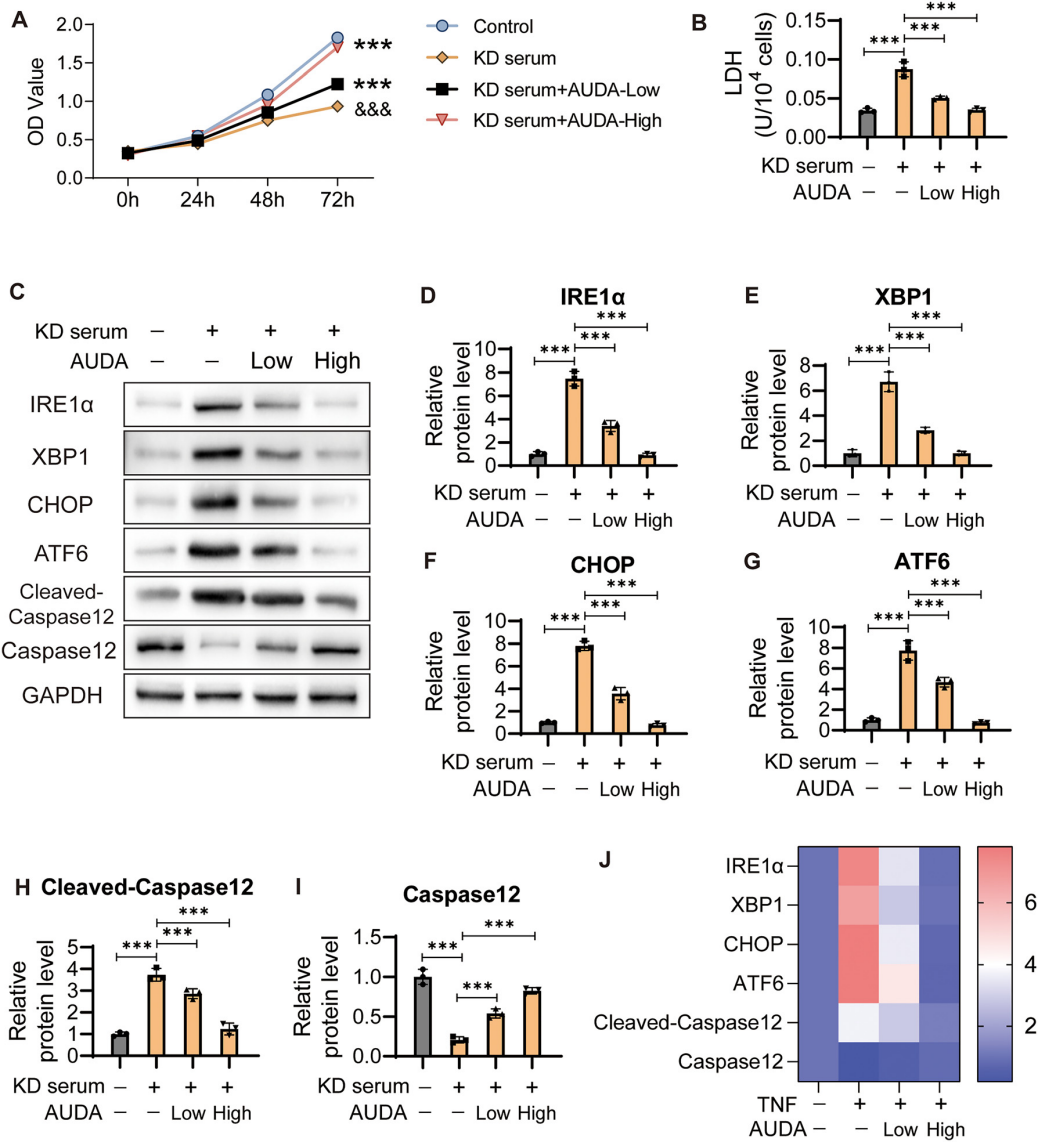
AUDA alleviates ERS induced by KD serum in HCAECs. (A) The HCAECs were cultured in medium with 15 % KD serum to generate KD cell model with healthy people serum as a control group. Then, HCAECs were treated with 50 μmol/L (low) and 100 μmol/L (high) AUDA for 24 h. The viability was detected using CCK8 assay. ***P < 0.001 versus control, &&&*P* < 0.001 versus KD serum treated cells. (B) The level of LDH was detected using Elisa assay. (C) The expression of ERS marker factors was detected. The protein levels of IRE1α (D), XBP1 (E), CHOP (F), ATF6 (G), Cleaved-Caspase12 (H) and Caspase12 (I) were standardized to GAPDH. (J) Heatmap of ERS marker factors expression. ***P < 0.001.

To further confirm our results, we collected serum samples from patient with KD and healthy individuals. HCAECs were treated with 15 % KD serum for 24 h. Cells treated with 15 % serum from healthy individuals were used as the control group. Cell viability and ERS markers were detected using CCK8 and Elisa assays. Similar results were observed in HCAECs treated with KD serum. The viability of HCAECs treated with KD serum was significantly lower that of control cells. After treatment with 50 μmol/L or 100 μmol/L AUDA, the viability significantly increased in KD serum-treated cells (Figure 3A). LDH level increased significantly in KD serum-treated cells and decreased markedly after treatment with 50 μmol/L or 100 μmol/L AUDA (Figure 3B). Similarly, the expression levels of IRE1α, XBP1, CHOP, ATF6 and Cleaved-Caspase12 were enhanced by KD serum treatment, and inhibited by 50 μmol/L or 100 μmol/L AUDA. In contrast, the Caspase12 expression was suppressed by KD serum treatment and promoted by 50 μmol/L or 100 μmol/L AUDA in HCAECs (Figure 3C–I).

### 3.4 AUDA inhibits KD serum induced apoptosis in HCAECs

**Figure 4:**
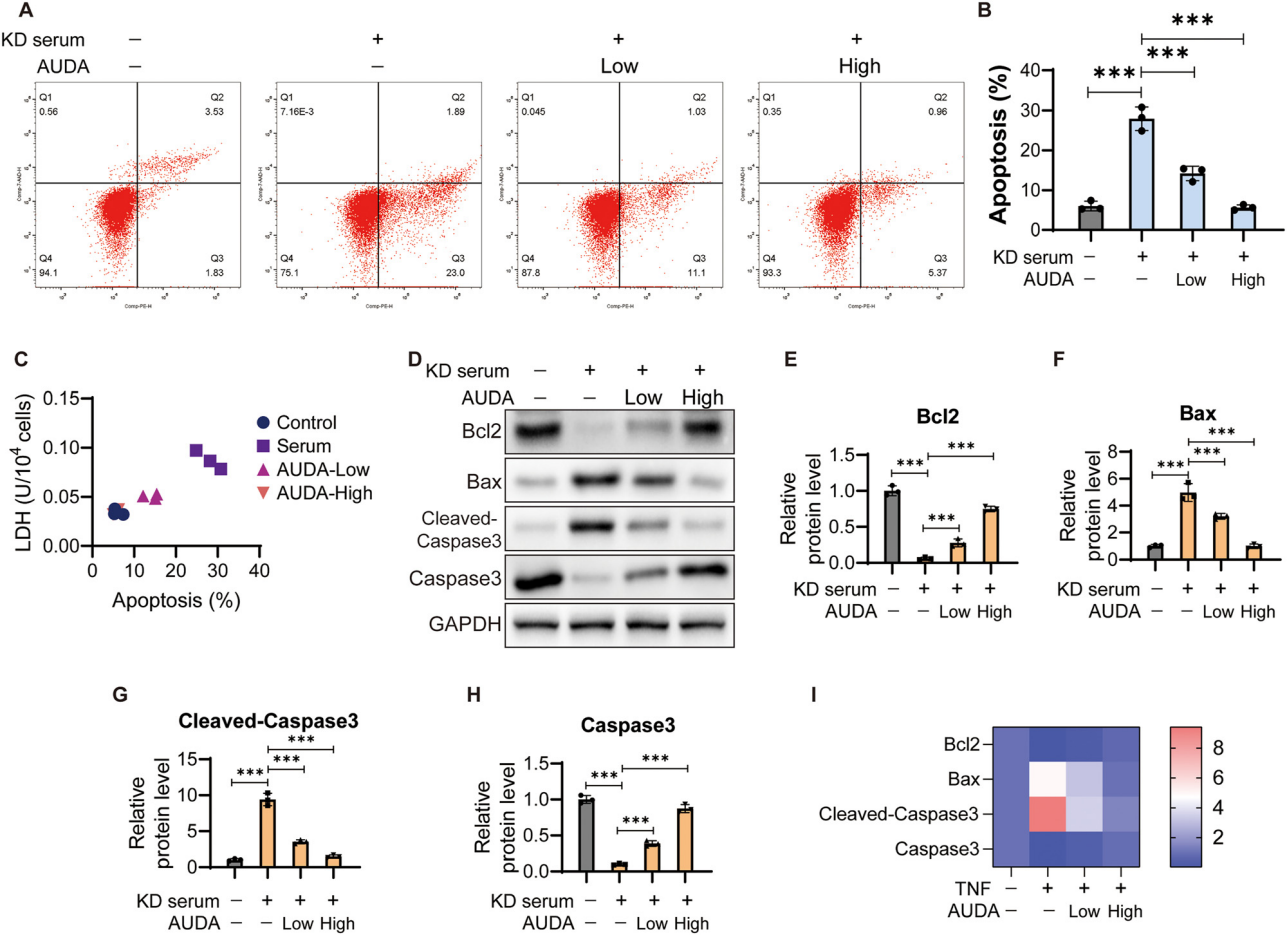
AUDA inhibits KD serum induced apoptosis in HCAECs. (A) Apoptosis was analyzed using flow cytometry. (B) Percentage of apoptotic cells was analyzed using FlowJo software. (C) The correlation between apoptosis and LDH. (D) Apoptotic protein expression was detected. The protein levels of Bcl2 (E), Bax (F), Cleaved-Caspase3 (G) and Caspase3 (H) were standardized to GAPDH. (I) Heatmap of apoptotic protein expression. ***P < 0.001.

Similar results were observed in HCAECs treated with KD serum. The percentage of apoptotic cells increased significantly in KD serum-treated cells, and declined significantly after AUDA treatment (Figure 4A and B). In addition, the trend in apoptosis was consistent with that of LDH across all groups (Figure 4C). Western blot results showed that KD serum increased the levels of Bax and Cleaved-Caspase3, and decreased the levels of Bcl2 and Caspase3 (Figure 4E–I). After treatment with AUDA for 24 h, Bax and Cleaved-Caspase3 expression was inhibited, and Bcl2 and Caspase3 expression were significantly promoted compared with the KD serum group cells (Figure 2E–I).

### 3.5 STAT1 enhancer 2-NP reverses the protective effect of AUDA on TNF-treated HCAECs

**Figure 5:**
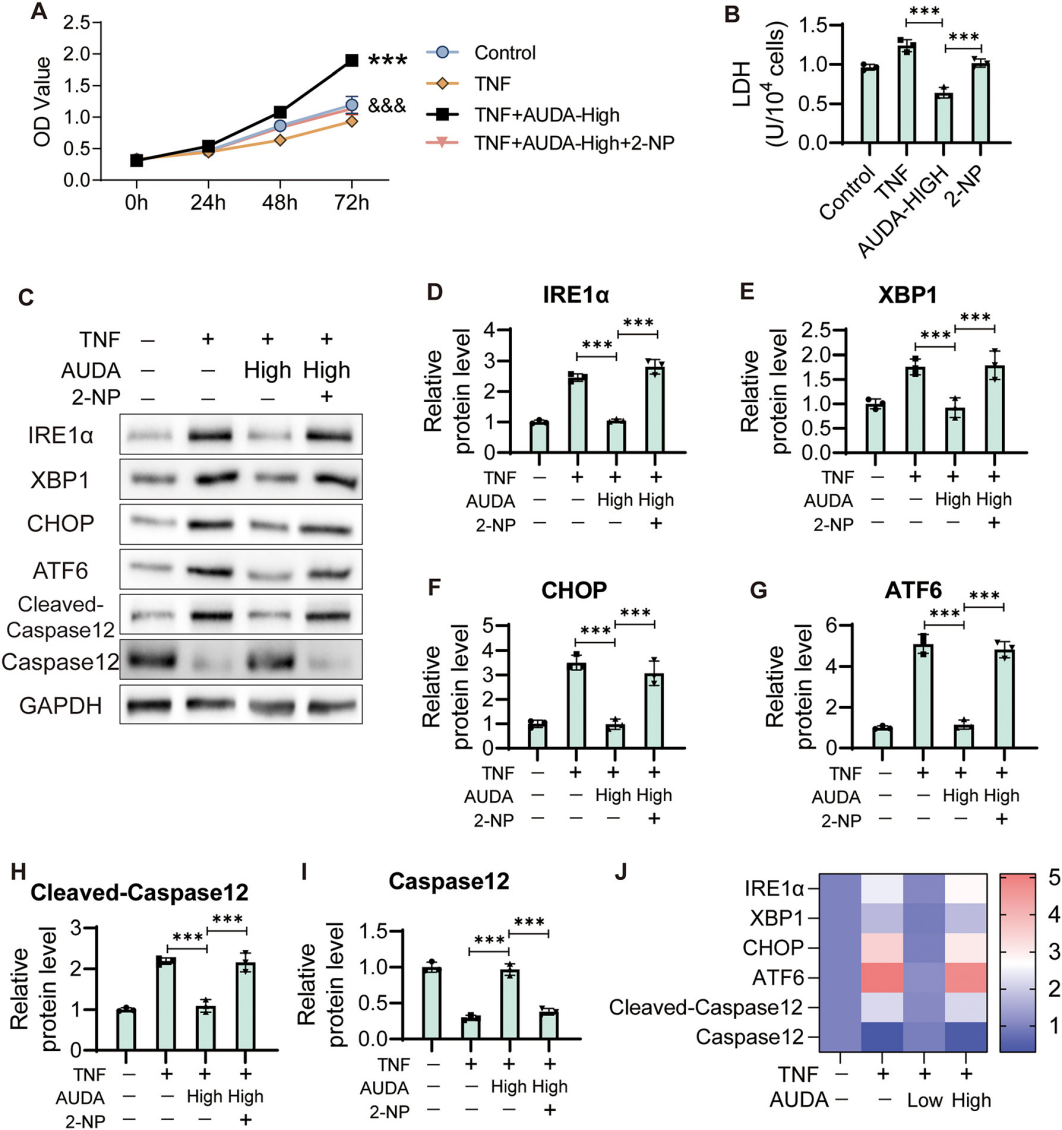
AUDA inhibits ERS by suppressing the JAK/STAT1 pathway. (A) 45 mmol/L of STAT1 transcription enhancer (2-NP) was used to activate the JAK/STAT1 signaling pathway. (B) Cell viability was detected using CCK8 assay. ***P < 0.001 versus TNF control, &&&*P* < 0.001 versus TNF and AUDA-treated cells. (B) The level of LDH was detected using Elisa assay. (C) The expression of ERS marker factors was detected. The protein levels of IRE1α (D), XBP1 (E), CHOP (F), ATF6 (G), Cleaved-Caspase12 (H) and Caspase12 (I) were standardized to GAPDH. (J) Heatmap of ERS marker factors expression. ***P < 0.001.

In previous studies, we confirmed that AUDA exerted an anti-inflammatory effect in HCAECs through STAT1 signaling pathway [4]. Here, we treated TNF induced HCAECs with STAT1 transcription enhancer (2-NP) to conduct a rescue experiment. As shown in Figure 5A, 2-NP inhibited the promoting effect of AUDA on cell proliferation in TNF treated HCAECs (Figure 5A). Similarly, 2-NP blocked the inhibitory effect of AUDA on LDH activity (Figure 5B). Western blot results showed that the inhibitory effect of AUDA on the expression levels of IRE1α, XBP1, CHOP, ATF6 and Cleaved-Caspase12 were rescued by 2-NP (Figure 5C–H). The promotion of Caspase12 expression induced by AUDA was also suppressed by 2-NP in TNF treated HCAECs (Figure 5I).

## 4 Discussion

The pathogenesis of KD remains unclear, and factors such as the environment, immunity, and genetics contribute to its occurrence [17], 18]. Clinical studies have found that coronary artery disease is related to endothelial cell dysfunction, and ERS-induced endothelial cell apoptosis [19], 20]. If effective interventions can be administered after early detection, coronary artery disease can be prevented.

In previous studies, we found that AUDA promotes endothelial cell growth, migration, and angiogenesis, and inhibits inflammation in the heart tissue of KD mice by activating the EETs-PPARγ signaling pathway [4], 5]. In the present study, we treated HCAECs with TNF and KD serum to further analyze the therapeutic effect of AUDA in KD by inhibiting ERS and apoptosis. In HCAECs induced by TNF and KD serum, cell viability was significantly reduced, while the cell death indicator LDH and the levels of IRE1α, XBP1, CHOP, ATF6 and Cleaved-Caspase12 were significantly increased. LDH is a key biomarker of cellular damage [21]. TNF induces the release of intracellular LDH into the cell culture supernatant by compromising plasma membrane integrity [16], 22].

Cell death/cytotoxicity, as a potential consequence of severe ERS, was evaluated by measuring lactate dehydrogenase (LDH) release. To specifically monitor ERS activation, we examined the expression of canonical markers such as GRP78/BiP and CHOP by Western blotting/qPCR.

Meanwhile, Caspase12 expression significantly decreased in TNF and KD serum-treated cells. Importantly, apoptosis increased significantly, and the apoptotic signaling pathway was activated in TNF and KD serum-treated cells. These results confirmed that TNF and KD serum treatment led to ERS and apoptosis in HCAECs. KD HCAECs were then treated with AUDA. Our results showed that even low levels of AUDA alleviated ERS and apoptosis induced by TNF or KD serum, as well as the inhibition of cell viability. Previous studies have shown that AUDA prevents diabetic cardiomyopathy by reducing apoptosis in mice [23]. AUDA has also been found to inhibit cerebral ischemic injury through vascular and neuroprotection [24]. AUDA has been identified as an effective treatment for vascular endothelial cell diseases.

Our previous research has shown that AUDA exerts anti-inflammatory effects by inhibiting the JAK/STAT1 signaling pathway in HCAECs [5]. Here, HCAECs were treated with STAT1 transcription enhancer 2-NP to perform the rescue experiment. After enhancing STAT1 transcription in AUDA-treated cells, cell activity declined to the control level. The cell death indicator LDH and the levels of IRE1α, XBP1, CHOP, ATF6 and Cleaved-Caspase12 increased significantly in the presence of the STAT1 transcription enhancer. In contrast, Caspase12 expression was inhibited by 2-NP. Activation of STAT1 blocked the inhibitory effect of AUDA on ERS and apoptosis in TNF treated HCAECs. These results indicated that AUDA suppressed ERS and apoptosis in HCAECs through the STAT1 signaling pathway. STAT1 is involved in the regulation of ERS in various cells, including brain [25], lung cells [26], and macrophages [27] thus participating in the regulation of various human diseases. As a clinical intervention target, STAT1 is extremely valuable in vascular lesions in patients with KD. Crucially, the fact that the STAT1 transcriptional enhancer (2-NP) abolishes the protective effects of AUDA strongly indicates that STAT1 is a key downstream mediator in this process. These findings not only shed light on mechanisms of KD-associated vasculitis but also position AUDA as a promising candidate for further therapeutic development. As the first-line therapy of KD, intravenous immunoglobulin (IVIG) exerts its effects through generalized immunomodulatory effects. However, a significant subset of patients does not respond adequately. In contrast, AUDA employs a more targeted strategy by directly reducing ERS and apoptosis in endothelial cells, which are key pathological events in KD vasculitis. Future studies could aim to evaluate the potential of AUDA in providing a complementary treatment option for IVIG-resistant patients by targeting downstream inflammatory and stress signals.

However, there were still several limitations in this study. Our findings were derived from *in vitro* models; therefore, essential *in vivo* studies are needed. Critical translational questions remain unaddressed. Importantly, these findings require validation through rigorously designed clinical trials to establish both the safety and efficacy of AUDA in children with KD. In addition, the specific molecular interaction between AUDA and STAT1 remains unclear. Specifically, it remains undetermined whether AUDA directly reduces STAT1 phosphorylation, impairs its nuclear translocation, or suppresses its transcriptional activity. Future studies are warranted to delineate the exact step in the STAT1 signaling that is targeted by AUDA.

In conclusion, AUDA inhibits TNF and KD serum-induced ERS and apoptosis in HCAECs. Combining previous research, AUDA treatment can alleviate cellular dysfunction in endothelial cells and prevent coronary artery injury in patients with KD.
